# Poor outcome of patients with COVID-19 after CAR T-cell therapy for B-cell malignancies: results of a multicenter study on behalf of the European Society for Blood and Marrow Transplantation (EBMT) Infectious Diseases Working Party and the European Hematology Association (EHA) Lymphoma Group

**DOI:** 10.1038/s41375-021-01466-0

**Published:** 2021-11-08

**Authors:** Anne Mea Spanjaart, Per Ljungman, Rafael de La Camara, Gloria Tridello, Valentín Ortiz-Maldonado, Alvaro Urbano-Ispizua, Pere Barba, Mi Kwon, Dolores Caballero, Pierre Sesques, Emmanuel Bachy, Roberta Di Blasi, Catherine Thieblemont, Friso Calkoen, Pim Mutsaers, Johan Maertens, Livia Giannoni, Emma Nicholson, Matthew Collin, Carlos Pinho Vaz, Elisabetta Metafuni, Joaquin Martinez-Lopez, Fiona L. Dignan, Josep-Maria Ribera, Arnon Nagler, Frantisek Folber, Robin Sanderson, Adrian Bloor, Fabio Ciceri, Nina Knelange, Francis Ayuk, Nicolaus Kroger, Marie José Kersten, Stephan Mielke

**Affiliations:** 1 Department of Hematology, Amsterdam University Medical Centers, Cancer Center Amsterdam and LYMMCARE, Amsterdam, The Netherlands; 2 Department of Cellular Therapy and Allogeneic Stem Cell Transplantation (CAST), Karolinska University Hospital Huddinge and Karolinska Comprehensive Cancer Center, Stockholm, Sweden; 3 Department of Medicine Huddinge, Karolinska Institutet, Stockholm, Sweden; 4 Department of Hematology, Hospital Universitario de La Princesa, Madrid, Spain; 5 Pediatric Hematology Oncology, Azienda Ospedaliera Universitaria Integrata, Verona, Italy; 6 Department of Hematology, Hospital Clínic, Barcelona, Spain; 7 Department of Hematology, Vall d’Hebron University Hospital, Vall d’Hebron, Barcelona, Spain; 8 Department of Hematology, Institute of Health Research Gregorio Marañon, Hospital G. Universitario Gregorio Marañon, Madrid, Spain; 9 Department of Hematology, Hospital Universitario de Salamanca, IBSAL, Salamanca, Spain; 10 Department of Hematology, Hospices Civils de Lyon, Lyon Sud Hospital, Lyon, Pierre-Bénite France; 11 Department of Hematology, Assistance Publique Hôpitaux de Paris—Hopital Saint-Louis, Paris, France; 12 Department of Stem cell Transplantation,Princess Maxima Centre for Paediatric Oncology, University Medical Centre Utrecht, Utrecht, The Netherlands; 13 Department of Hematology, Erasmus MC Cancer Center, Rotterdam, The Netherlands; 14 Deptartment of Hematology, University Hospital Gasthuisberg, Leuven, Belgium; 15 Ematologia e Centro Trapianti, IRCCS Ospedale Policlinico San Martino, Genova, Italy; 16 Department of Haematology, The Royal Marsden Hospital, London, UK; 17 Adult HSCT Unit, Northern Centre for Bone Marrow Transplantation, Newcastle Tyne, UK; 18 BMT Unit, Inst. Português de Oncologia do Porto, Porto, Portugal; 19 Dipartimento di Diagnostica per Immagini, Radioterapia Oncologica ed Ematologia, Fondazione Policlinico Universitario A. Gemelli IRCCS, Roma, Italy; 20 Department of Hematology, Hospital Univ. 12 de Octubre, CNIO, Complutense University, Madrid, Spain; 21 Clinical Haematology Department, Manchester Royal Infirmary, Manchester, UK; 22 Clinical Hematology Department, Catalan Institute of Oncology, Hospital Germans Trias i Pujol, Josep Carreras Research Institute, Barcelona, Spain; 23 Chaim Sheba Medical Center, Tel Aviv University, Tel HaShomer, Tel Aviv-Yafo, Israel; 24 Department of Internal Medicine, Hematology and Oncology, University Hospital Brno, Brno, Czechia; 25 Department of Haematological Medicine, Kings College Hospital, London, UK; 26 Adult Leukaemia and Bone Marrow Transplant Unit, Christie NHS Trust Hospital, University of Manchester, Manchester, UK; 27 Hematology and BMT Unit, IRCCS San Raffaele Scientific Institute, Milan, Italy; 28 Dept. of Medical Statistics & Bioinformatics, EBMT Data Office, Leiden, The Netherlands; 29 Department of Stem Cell Transplantation, University Hospital Eppendorf, Hamburg, Germany; 30 Department of Laboratory Medicine, Karolinska Institutet, Stockholm, Sweden

**Keywords:** Infectious diseases, Haematological cancer

## To the Editor

COVID-19 is posing a significant threat to health in vulnerable patients, such as immunocompromised patients. For hematopoietic cell transplantation (HCT) recipients and patients with hematologic malignancies it is known that COVID-19 leads to severe morbidity and high mortality as compared to the general population [1–3]. For patients treated with Chimeric Antigen Receptor T-cell (CAR-T-cell) therapy for B-cell malignancies however, descriptions of the clinical course and outcome are still limited to small case series and case reports [4–8]. CAR-T-cell therapy recipients are believed to be at high risk of poor outcomes from COVID-19 due to their severely immunocompromised state, caused by prior lymphodepleting immunochemotherapy and CAR-T-cell therapy related side effects such as B-cell depletion, hypogammaglobulinemia, and cytopenias. In order to rapidly inform the medical field on the impact of COVID-19 on CAR-T-cell therapy recipients, the EBMT Infectious Diseases Working Party and the EHA Lymphoma Group joined forces and present the clinical course of COVID-19 in the largest European cohort to date.

In response to the COVID-19 pandemic, the EBMT developed a special report form to conduct a multicenter survey study on patients with COVID-19 after CAR-T-cell therapy for hematologic malignancies (Fig. S1). Only PCR positive SARS-CoV-2 diagnosed patients with at least 6 weeks follow-up at the database lock on June 1st, 2021 were included. Duration of disease was defined as time from COVID-19 diagnosis to resolution of disease, defined as a negative PCR test or resolution based on clinical findings, or death. Descriptive statistics were used for clinical characteristics. Overall survival was estimated using the Kaplan–Meier method, considering death due to any cause as an event and time from COVID-19 infection to the last date of follow-up as survival time. Univariate and multivariate risk factor analyses for overall survival were performed using the Cox regression model. Variables with a *p* value <0.1 at univariate analysis were entered into the multivariate model and selected according to a stepwise selection.

A total of 57 patients from 11 countries were reported to the EBMT (Table S1). One patient with incomplete data at diagnosis and without any follow-up information had to be excluded from the analysis. The median age of these 56 patients was 57.7 years, including 55 adults and one child. Thirty-two patients were male. In the majority of patients CAR-T-cell therapy was given for B-cell-non-Hodgkin lymphoma (82.1%) and most patients either were in complete remission after CAR-T-cell therapy (62.5%) or had a partial response (12.5%) (Table 1). The median time from CAR-T-cell infusion to COVID-19 diagnosis was 7.4 months (min–max 1 day—25.3 months). In 87.5% of patients symptoms were reported including fever (64.3%), upper respiratory complaints (51.8%), cough (57.1%), fatigue (39.3%), myalgia/arthralgia (17.9%), and vomiting/diarrhea (16.1%) (Table S1). Most patients (82.1%) had a Lansky/Karnofsky-score above 70 points indicating they were still able to care for themselves. Eighteen patients (32.1%) had metabolic comorbidities (Table 1). Forty-five patients (80%) had to be admitted to hospital for COVID-19 related symptoms, with a median duration of 25 days and 24 patients needed oxygen support (42.9%). Twenty-two (39.3%) patients were admitted to the intensive care unit (ICU) for a median duration of 14 days and 16 of these patients (72.7%) needed mechanical ventilation. Many different treatments were given (Table 1) but most patients received convalescent plasma, steroids, or remdesivir. Additional clinical characteristics (e.g., CAR-T-cell therapy, prior HCT, and laboratory results) can be found in Table S1.

**Table 1:** Baseline demographics and clinical characteristics of patients infected by SARS-CoV-2 and association between these characteristics and mortality.

Characteristic	Number (proportion) unless specified otherwise
Age, median age in years, (min–max)	57.7 (5.2–72.8)
Male sex	32 (57.1)
CAR T-cell therapy indication	
B-cell acute lymphoblastic leukemia	7 (12.5)
B-cell non-Hodgkin lymphoma	46 (82.1)
Multiple myeloma	3 (5.4)
Disease status at time of COVID-19 diagnosis	
Relapse/progression	14 (15)
Partial response	7 (12.5)
Complete remission	35 (62.5)
Metabolic comorbidities *(obesity, hypertension, diabetes, hypercholesterolemia, smoking,* and *cardiovascular disease)*	
Yes	18 (32.1)
No	38 (67.9)
Lansky/Karnofsky-score at time of COVID-19 diagnosis	
<70	10 (17.9)
≥70	46 (82.1)
Oxygen therapy	
No oxygen support needed,	32 (57.1)
Oxygen support needed	24 (42.9)
Invasive mechanical ventilation	16 (28.6)
Noninvasive ventilation *(noninvasive positive pressure ventilation)*	2 (3.6)
COVID-19 treatment	
Dexamethasone	10 (17.9)
(Methyl)prednisolone	2 (3.6)
Tocilizumab	8 (14.3)
Baricitinib	2 (3.6)
Eculizumab	1 (1.8)
Remdesivir	20 (35.7)
Lopinavir/Ritonavir	1 (1.8)
Chloroquine/Hydroxychloroquine	1 (1.8)
Convalescent plasma	17 (30.4)
IVIG non-SARS-CoV-2	1 (1.8)
Anticoagulants *(either prophylaxis or treatment)*	19 (33.9)
No treatment	15 (26.8)
Resolution of COVID-19 in surviving patients	
Clinical resolution	10 (17.9)
Virologic (PCR) resolution	21 (37.5)
Clinical COVID-19 median duration, days (min–max)	20.0 (0–157)
Virologic COVID-19 median duration, days (min–max)	48 (6–113)
Factors associated with mortality (HR, 95% CI, *P* value)	
Age at time of COVID-19	1.39 (1.05–1.86), *p* = 0.02
Performance status at time of COVID-19	0.71 (0.56–0.89), *p* = 0.003
Metabolic comorbidity	2.75 (1.19–6.35), *p* = 0.02
Tumor not in complete remission at time of COVID-19	2.40 (1.04–5.55), *p* = 0.04

**Fig. 1:**
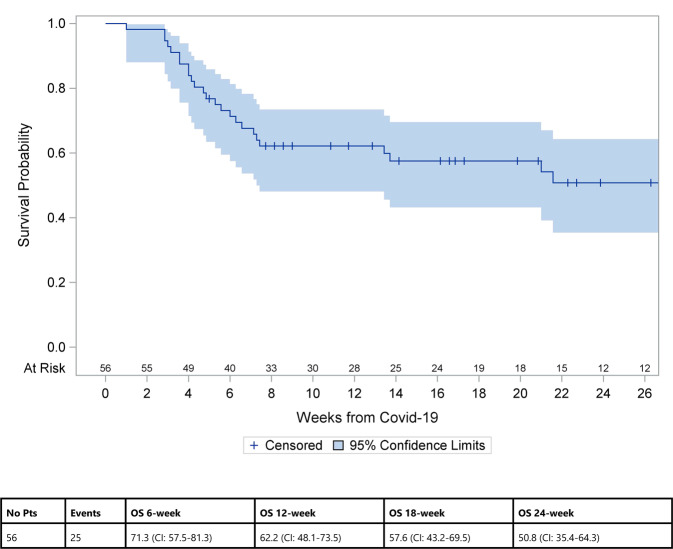
Overall survival of patients after COVID-19 diagnosis. Overall survival was estimated using the Kaplan–Meier method, considering death due to any cause as an event and time from COVID-19 infection to the last date of follow-up as survival time.

At the time of analysis, 25 of 56 patients had died (44.6%), the vast majority (23 of 25) due to COVID-19, resulting in a COVID-19 attributable mortality rate of 41.1%. The median age was 60.3 years. Twelve patients were in complete remission at time of death, four had a partial response and nine had refractory/relapsed disease. Median time from COVID-19 until death was 4.9 weeks (min–max 1–21.6). The Kaplan–Meier estimate of overall survival is shown in Fig. 1. In univariate analysis (Table S2) significant factors associated with mortality risk were older age, worse performance status, metabolic comorbidities and not being in complete remission at time of COVID-19 diagnosis. In multivariate analysis (Table 1), older age (10-year-effect, HR 1.39, 95% CI 1.05–1.86, *p* = 0.02), not being in complete remission at time of COVID-19 diagnosis (HR 2.40, 95% CI 1.04–5.55, *p* = 0.04) and having metabolic comorbidities (HR 2.75, 95% CI 1.19–6.35, *p* = 0.02) were associated with a higher mortality risk and better performance status (10 point effect, HR 0.71, 95% CI 0.56–0.89, *p* = 0.003) with a lower mortality risk. Sex, time from CAR-T-cell therapy to COVID-19 diagnosis and the occurrence of neurotoxicity or cytokine release syndrome after CAR-T-cell infusion did not have a significant effect on mortality in the multivariate analysis. The median time to clinical resolution of COVID-19 was 20 days (min–max 0–157) and the median time to virologic (PCR) resolution 48 days (min–max 6–113). The number of patients in our cohort study was probably too small to observe any effect of treatment on outcome. We did observe a positive effect of convalescent plasma on overall survival in univariate analysis when only looking at the 45 patients who were admitted to hospital (HR 0.37 CI 0.15–0.93, *p* = 0.03) but this effect was not confirmed in a multivariate model. These findings from regression analysis are considered exploratory and need to be validated in a larger cohort.

Our results illustrate that patients diagnosed with COVID-19 after B-cell-targeted CAR-T-cell therapy have a very poor outcome. The COVID-19 attributable mortality rate of 41% is at least 4 but mostly 10–40 times higher than reported in the general population of countries currently most affected by COVID-19 (Mortality Analyses—Johns Hopkins Coronavirus Resource Center (jhu.edu), August 2021). These results are in line with previous studies reporting on poor outcomes in patients with hematologic malignancies. Vijenthira et al. have shown a mortality rate of 34% in a meta-analysis of 3377 patients with hematologic malignancies. In the subgroup of 485 lymphoma patients mortality was 32% and older age, comorbidity and progressive disease were associated with mortality in multivariate analysis [1]. In a multicenter study of 177 lymphoma patients, being on active treatment or higher number of prior therapy lines was not associated with mortality, supporting the advice not to withhold treatment from patients [9] Another cohort study of 111 lymphoma patients however showed that treatment with anti-CD20 monoclonal-antibodies in the past 12 months was associated with an increased risk of death and longer duration of COVID-19 symptoms [8].

Patients with hematologic malignancies also have an increased risk of mortality relative to patients with solid tumors (around 1.7 times higher), independent of potential confounding effects such as performance status. This may in part be due to impairment of B-cells and SARS-CoV-2-specific antibody responses seen in patients with hematologic malignancies whereas in patients with solid tumors humoral and cellular immune responses are similar to patients without cancer, as was shown by Bange et al. [10]. They also showed that patients with T-cell depletion have the highest risk of death independent of cancer subtype or presence of B-cell responses, whereas patients with hematologic malignancies and higher numbers of CD8 cells have more often detectable SARS-CoV-2-specific T-cell responses and improved survival. These findings shed valuable insight into why severely lymphodepleted CAR-T-cell therapy recipients, even independently of their remission status have probably such a high mortality rate. Limitations of our study include the lack of data on B- and T-cell numbers.

Besides the high risk of death when faced with COVID-19, immunocompromised patients also have to endure long duration of severe symptoms often requiring prolonged hospital admission [8]. In our cohort, the median hospital admission duration was 25 days with a maximum up to 171 days and almost half of the admitted patients had to be admitted to the ICU for a median duration of 14 days. Additionally, patients with hematologic malignancies, especially when treated with anti-CD20 monoclonal-antibodies, have lower rates of seroconversion and prolonged viral shedding after COVID-19 (mean 61 days) compared to patients with solid tumors [11]. Prolonged viral shedding of viable SARS-CoV-2 for at least 2 months has been described after HCT and CAR-T-cell therapy [4]. In our cohort, all alive patients had resolution of COVID-19 symptoms but only 21 of 31 patients were retested with PCR and the other 10 patients had resolution of disease based on clinical findings. We were unable to retrieve information on longitudinally obtained PCR Ct values or viral cultures. It is concerning that more reports appear describing the evolution of multimutational SARS-CoV-2 variants in immunocompromised patients with long lasting COVID-19 that might be more transmissible and pathogenic [12]. Longer isolation of CAR-T-cell recipients until viral shedding has ended or until negative PCR results are obtained, should be considered. There are currently no data on early treatment of SARS-CoV-2 infection in CAR-T-cell recipients with convalescent plasma, COVID-19 hyperimmune globulin or SARS-CoV-2 monoclonal antibodies and/or on using these therapeutic modalities in patients with a protracted COVID-19 disease course However, there is a clinical rationale to use a cocktail of monoclonal antibodies based on results of randomized clinical trials performed in the general population, with the greatest benefit observed in seronegative patients [13].

It is clear that preventing SARS-CoV-2 transmission and disease development in CAR-T-cell recipients it of the utmost importance. Unfortunately, preliminary results show that patients with B-cell depletion following rituximab or B-cell directed CAR-T-cell treatment are also less likely to achieve effective antibody responses to COVID-19 vaccination [14, 15]. Results of cellular responses to vaccination in this group are currently lacking. This implies that as long as it remains uncertain whether currently applied vaccination strategies are effective in CAR-T-cell therapy recipients, vaccination of health-care personnel, and family members in combination with protective measures against viral exposure are crucial in protecting this vulnerable group of patients. An absolute downside of these protective measures is their negative impact on social participation and quality of life and better prevention and treatment strategies are therefore urgently needed.

## Supplementary information

Supplementary data
