# Supplementary material for: Poor outcome of patients with COVID-19 after CAR T-cell therapy for B-cell malignancies: results of a multicenter study on behalf of the European Society for Blood and Marrow Transplantation (EBMT) Infectious Diseases Working Party and the European Hematology Association (EHA) Lymphoma Group
Source: Leukemia. 2021 Nov 8;35(12):3585–8. doi: 10.1038/s41375-021-01466-0 (PMC8573311; doi:10.1038/s41375-021-01466-0)
Supplement: Supplementary file 1 — Supplementary data [file 41375_2021_1466_MOESM1_ESM.pdf]

SUPPLEMENTARY DATA

Title

Poor outcome of patients with COVID-19 after CAR T-cell therapy for B-cell malignancies: Results of a multicenter study on behalf of the European Society for Blood and Marrow Transplantation (EBMT) Infectious Diseases Working Party and the European Hematology Association (EHA) Lymphoma Group

Supplementary Figure 1. COVID-19 clinical report form

Impact of COVID-19 in CAR-T cell recipients

EBMT-IDWP

Non-Interventional Prospective Study

Patients diagnosed **before or on December 31st 2020** can be included if **tested positive by PCR**

Patients diagnosed **after December 31st 2020** can be included if **tested positive by PCR or antigen test**

CRF

STUDY PERIOD

FROM MARCH 1ST 2020

UPDATED ON 15-02-2021

Patient Identification

Centre number (CIC) _______________
Hospital ____
City / Country ____
Contact person ____
Contact person e-mail ____

Patient Unique Identification Code (UIC) _______________
Hospital Unique Patient Number (UPN) _______________
Date of birth _____________________ *(yyyy-mm-dd)*

Sex
☐ male
☐ female

Date of this report _____________________ *(yyyy-mm-dd)*

Previously registered to the EBMT COVID-19 study
☐ Yes
☐ No: please complete the registration form

<u>This form should be completed 6 weeks after covid-19 diagnosis OR if the patient is deceased. If the patient is unresolved and alive after 6 weeks/at completion of this report, please update us again 12 weeks after diagnosis</u>

NOTE: If your country/center requires an ethical permit to submit this type of data, please, leave UIC and UPN blank and only give year of birth and date and month of transplant. We can then go back later after correct permits have been obtained

Basic CAR-T data

CAR-T cell type (target antigen)
☐ CD-19
☐ BCM
☐ Other

Costimulatory molecule
☐ 41-BB
☐ CD28
☐ CD28 and 41BB
☐ Other

Infusion date ______________________ *(yyyy-mm-dd)*

Previous SCT
☐ allogeneic
☐ autologous
☐ None

Previous transplant date ______________________ *(yyyy-mm-dd)*

Baseline disease data

1. **Date of initial diagnosis** ______________________ *(yyyy-mm-dd)*

2. **Primary disease diagnosis (for which the CAR-T cells was given)**

☐ Acute Leukaemia
  ☐ Myelogenous (AML)
  ☐ Lymphoblastic (old ALL)
☐ Chronic Leukaemia
  ☐ Chronic Lymphocytic Leukaemia
☐ Lymphoma
  ☐ Non Hodgkin
  ☐ Hodgkin's Disease
☐ Myeloma /Plasma cell disorder
☐ Other diagnosis,
specify:_______________________________________

3. **Disease status before SARS-CoV-2 diagnosis:**

☐ Complete remission
☐ Partial remission
☐ Relapse
☐ Progression
☐ Refractory disease
☐ 1st line therapy

4. Date of last course of prior therapy ____________________ *(yyyy-mm-dd)*

Date of lymphodepletion ____________________ *(yyyy-mm-dd)*

Type of lymphodepletion (specify) ____

Cytokine release syndrome before COVID-19
☐ Yes; please give max grade (EBMT/ISCT) ________
☐ No
☐ Unknown

Neurotoxicity before COVID-19
☐ Yes; if yes, please, give max grade (EBMT ISCT) _________
☐ No
☐ Unknown

5. Performance status: Karnofsky/Lansky at the time of SARS-CoV-2 diagnosis

**Karnofsky status**	**Lansky Scale**<br>**(recipient age ≥ 1 year and <16 years)**	**Grade**
☐ Normal, no complaints	Fully active	**100**
☐ Able to carry on normal activities. Minor signs or symptoms of disease	Minor restriction in physically strenuous play	**90**
☐ Normal activity with effort	Restricted in strenuous play, tires more easily, otherwise active	**80**
☐ Care for self. Unable to carry on normal activity or to do active work	Both greater restrictions of, and less time spent in active play	**70**
☐ Requires occasional assistance, but able to care for most of his/her needs	Ambulatory up to 50% of time, limited active play with assistance/supervision	**60**
☐ Requires considerable assistance and frequent medical care	Considerable assistance required for any active play, fully able to engage in quiet play	**50**
☐ Disabled. Requires special care and assistance	Able to initiate quite activities	**40**
☐ Severely disabled. Hospitalization indicated though death nonimminent	Needs considerable assistance for quiet activity	**30**
☐ Very sick. Hospitalization necessary. Active supportive treatment necessary	Limited to very passive activity initiated by others (e.g., TV)	**20**
☐ Moribund	Completely disabled, not even passive play	**10**

Comorbidities at the time of COVID-19

6. Comorbidities

Smoker (current)	☐ No	☐ Yes
Smoker (former)	☐ No	☐ Yes
Alcohol abuser	☐ No	☐ Yes
Narcotics	☐ No	☐ Yes
Dyslipidemia	☐ No	☐ Yes
High blood pressure	☐ No	☐ Yes
Cardiovascular	☐ No	☐ Yes, specify ________________________________
Secondary malignancy	☐ No	☐ Yes, specify ________________________________
Other, specify	_____________________________	

Biological and clinical variables at time of COVID-19 episode

All biological and clinical variables should be recorded at the time of screening for SARS-CoV-2/time of diagnosis

8. Blood levels at the time of screening for SARS-CoV-2/time of diagnosis

	Value + unit *(if different from stated unit)*
Absolute neutrophil count *($x10^9/L$)*	
Absolute lymphocyte count *($x10^9/L$)*	
Total platelet count *($x10^9/L$)*	
Creatinine levels *(mg/dl)*	
C reactive protein levels *(mg/L)*	
LDH levels	
IgG level	

9. Bronchiolitis obliterans syndrome (BOS) <u>before</u> Covid-19

☐ No
☐ Yes
☐ Unknown

11. Other lung pathology for example BOOP

☐ No
☐ Yes, specify ____
☐ Unknown

12. Corticosteroid therapy for other reasons than covid-19

☐ No

☐ Unknown

☐ Yes, specify type and dose (mg/d)

☐ Prednisone ___________

☐ Methylprednisolone ___________

☐ Dexamethasone ___________

☐ Other ________________________

13. Immunosuppressant drug(s) within 2 months prior to and after the covid-19 episode

☐ Unknown

Drug name

14. Vitamin D levels in the 3 months <u>prior</u> to SARS-CoV-2 diagnosis *(if multiple levels are available, please note the most recent one)*

☐ unknown/not available

☐ available: 25-hydroxycholecalciferol _____ng/mL or __________nmol/L

1,25-dihydroxycholecalciferol ______ in pg/mL or ___________ in pmoll/L

Clinical signs/symptoms at SARS-CoV-2 diagnosis

15. Clinical signs/symptoms recorded during SARS-CoV-2 episode

Asymptomatic	☐ No	☐ Yes	☐ Unknown
Fever	☐ No	☐ Yes, date ____________________ *(yyyy-mm-dd)*	☐ Unknown
Upper respiratory symptoms	☐ No	☐ Yes, date ____________________ *(yyyy-mm-dd)*	☐ Unknown
Rhinorrea/ nasal congestion	☐ No	☐ Yes	☐ Unknown
Sinusitis	☐ No	☐ Yes	☐ Unknown
Taste disturbance	☐ No	☐ Yes	☐ Unknown
Smell disturbance	☐ No	☐ Yes	☐ Unknown
Otitis	☐ No	☐ Yes	☐ Unknown
Pharyngitis and/or tonsillitis	☐ No	☐ Yes	☐ Unknown
Cough	☐ No	☐ Yes	☐ Unknown
Sputum production	☐ No	☐ Yes	☐ Unknown

Fatigue	☐ No	☐ Yes	☐ Unknown
Myalgia or arthralgia	☐ No	☐ Yes	☐ Unknown
Diarrhoea	☐ No	☐ Yes	☐ Unknown
Vomiting	☐ No	☐ Yes	☐ Unknown
Conjunctivitis	☐ No	☐ Yes	☐ Unknown
Oxygen requirement to maintain oxygen sat >92%	☐ No	☐ Yes	☐ Unknown

16. Please specify where the patient was attended during the SARS-COV-2 infection

☐ Outpatient

☐ Hospitalised**,** date of admission ____________________ *(yyyy-mm-dd)*

17. Was this hospitalisation related to SARS-COV-2 infection?

☐ No

☐ Yes

18. Intensive care unit admission

☐ No

☐ Yes, date of admission ____________________ *(yyyy-mm-dd)*

date of discharge ____________________ *(yyyy-mm-dd)*

19. Ventilation:

☐ Use of non-invasive ventilation

☐ Use of invasive ventilation

☐ No need for ventilation

20. Use of high-flow oxygen therapy

☐ No

☐ Yes

☐ Unknown

Microbiological data

21.Was antigen testing performed?

☐ No

☐ Yes, date of test ____________________ *(yyyy-mm-dd)*

☐ positive for SARS-CoV-2

☐ negative for SARS-CoV-2

22.Was PCR test performed?

☐ No

☐ Yes, date of test ____________________ *(yyyy-mm-dd)*

☐ positive for SARS-CoV-2

☐ negative for SARS-CoV-2

23. PCR technique used (commercial PCR data) for covid-19/SARS-COV-2 and CARVs. Describe PCR platform (manufacturer) if known

____

24. Virus/es detected in samples from URT secretions (mark all typers and subtypes of CARVs detected in the sample/episode)

SARS-CoV-2	☐ positive	☐ negative	☐ not tested
Coronavirus 229E	☐ positive	☐ negative	☐ not tested
Coronavirus HKU1	☐ positive	☐ negative	☐ not tested
Coronavirus NL63	☐ positive	☐ negative	☐ not tested
Coronavirus OC43	☐ positive	☐ negative	☐ not tested
Influenza A type____________	☐ positive;	☐ negative	☐ not tested
Influenza B	☐ positive	☐ negative	☐ not tested
Human metapneumovirus	☐ positive	☐ negative	☐ not tested
Human parainfluenza virus 1	☐ positive	☐ negative	☐ not tested
Human parainfluenza virus 2	☐ positive	☐ negative	☐ not tested
Human parainfluenza virus 3	☐ positive	☐ negative	☐ not tested
Human parainfluenza virus 4	☐ positive	☐ negative	☐ not tested
Respiratory syncytial virus	☐ positive	☐ negative	☐ not tested
Enterovirus	☐ positive	☐ negative	☐ not tested
Rhinovirus	☐ positive	☐ negative	☐ not tested
Enterovirus/rhinovirus (EvRh)	☐ positive	☐ negative	☐ not tested
Adenovirus	☐ positive	☐ negative	☐ not tested
Human Bocavirus	☐ positive	☐ negative	☐ not tested
Other__________________	☐ positive	☐ negative	☐ not tested

25. Was a variant of SARS-CoV-2 detected?

☐ Unknown
☐ No

☐ Yes, please specify:

☐ B.1.1.1.7 (British variant)
☐ B.1.351 (South African variant)
☐ P.1 (Brazilian variant)
☐ CAL.20C (Southern Californian variant)
☐ Other ___________________________

26.Bronchoalveolar (BAL) performed
☐ No
☐ Yes, date of test ____________________ *(yyyy-mm-dd)*

27. BAL findings (describe significant findings only)

☐ Bacteria (describe all types and subtypes of bacteria detected following standard nomenclature)____

____

☐ Virus/es (describe all types and subtypes of CARVs detected following standard nomenclature, including CMV and several other tested viruses). If CMV was found, please, give viral load.____

☐ Fungi including P Jirovecii (describe all types and subtypes of fungus detected following standard nomenclature, as well as galactomannan >1)

____

28. **Co-infection (mark according microbiological findings)**

☐ None

☐ Other virus than SARS-CoV-2
☐ Bacteria
☐ Fungal
☐ Combination, specify ____

COVID-19 treatment

28. Antiviral drugs used
☐ **None**

Drug name	Dose/schedule	Start date	End date
☐ Remdesvir			
☐ Lopenavir/ritonavir			
☐ Favipavir			
Other ______________________			
Other ______________________			
Other ______________________			

29. Anti-coagulation agents

☐ **None**

Drug name	Dose/schedule	Start date	End date
☐ Low Molecular Weight Heparin			
☐ Unfractionated heparin			
☐ Fondaparinux			
☐ Rivaroxaban			
☐ Dabigatran			
☐ Apixaban			
☐ Edoxaban			
☐ Acenocumarol			
☐ Warfarin			
Other ________________________			
Other ________________________			
Other ________________________			

30.Anti-inflammatory drugs for treatment of COVID-19 for example IL-6 receptor inhibitors; corticosteroids, colchicine, etc)

☐ **None**

Drug name	Dose/schedule	Start date	End date
☐ Tozilizumab			
☐ Siltuximab			
☐ Ruxolutinib			
☐ Anakinra			
☐ Baricitinib			
☐ Eculizumab			
☐ Colchicine			
Other ________________________			
Other ________________________			
Other ________________________			

31. Immune or cellular therapies given for covid-19 episode

☐ unknown

☐ no

☐ yes, please specify:

☐ immunoglobulins, date ____________________ *(yyyy-mm-dd)*

☐ convalescent plasma, date ____________________ *(yyyy-mm-dd)*

☐ anti-SASRS-CoV-2 monoclonal antibodies, date ____________________ *(yyyy-mm-dd)*

☐ cellular therapy, specify type date ____________________ *(yyyy-mm-dd)*

☐ mesenchymal cells

☐ other ___________________________

☐ unknown

Vaccination

31. Did this patient receive a covid-19 vaccine? *(before or after the SARS-CoV-2 infection/COVID-19 episode)*

☐ unknown, continue to Q35

☐ no, please specify reason and continue to Q35

☐ not yet available

☐ patient declined

☐ decided by physician due to current disease status

☐ other, specify ____

☐ Yes, please specify in Q34.

32. Vaccination brand and date

Vaccine brand	First Vaccine/dose	Second Vaccine/dose	Third Vaccine/dose
	Date ___________	Date ___________	Date ___________
Pfizer/BioNTech COVID-19 mRNA Vaccine NT162b2	☐	☐	☐
AstraZeneca/Oxford COVID-19 AZD1222	☐	☐	☐
Moderna COVID-19 Vaccine (mRNA-1273)	☐	☐	☐
Sputnik V by Gamaleya Research Institute	☐	☐	☐
J&J JNJ-78436735	☐	☐	☐
CureVac CVnCoV mRNA Vaccine	☐	☐	☐
Other ____________________	☐	☐	☐

33. Did the patient receive an influenza vaccine in the 12 months prior to SARS-CoV-2 diagnosis?

☐ unknown

☐ no

☐ yes, date ____________________ *(yyyy-mm-dd)*

Clinical data and outcome

34. Pulmonary radiological findings

☐ Unknown

☐ No

☐ Yes, describe type of radiology and pulmonary pattern

____

35. Upper respiratory tract disease (combination of upper respiratory symptoms (i.e. rhinorrhea, sinusitis, otitis, or pharyngitis) and detection of COVID-19 in the upper RT)

☐ Yes

☐ No

☐ Unknown

36. Lower RTD

☐ Possible

☐ Probably

☐ Proven

Lower respiratory tract disease category
**Possible:** detection of a covid-19 in the upper respiratory tract **with** clinical symptoms of tracheitis, bronchitis, bronchiolitis, or pneumonia (new onset of cough, rales, wheezing, cough related chest pain, shortness of breath, dyspnea, or hypoxia) **in conjunction with** the identification of new pulmonary infiltrates by chest X-ray or thoracic CT scan.

37. Covid-19 resolution

☐ Unknown

☐ Unresolved, date last checked ____________________ *(yyyy-mm-dd)*

☐ Resolution, date ____________________ *(yyyy-mm-dd)*

☐ Resolution (clinical if no PCR available), date ____________________ *(yyyy-mm-dd)*

38. Date of hospital discharge ____________________ (yyyy-mm-dd)

39. Date of last follow-up ____________________ *(yyyy-mm-dd)*

40. Primary disease relapse status at last follow-up

☐ Complete remission

☐ Partial remission

☐ Relapse, date of first ____________________ *(yyyy-mm-dd)*

☐ Progression

☐ Refractory disease

41. Status at last follow-up

☐ Alive

☐ Dead

42. If dead, cause of death (tick all that apply)

☐ COVID-19 (unresolved)

☐ COVID-19 (resolved)

☐ relapse

☐ other (describe all attributable causes of death) ______________________________________

Comments

____

Thank you!!!!

Please send the completed form to:
Nina Knelange
EBMT Data Office Leiden / IDWP

Fax +49 711 4900 8723 / +49 180 500 290 623
E-mail: idwpebmt@lumc.nl

Supplementary Table 1. clinical characteristics of patients infected by SARS-Cov-2

**Characteristic**	**Number (Proportion)**
Country of patient	
Italy	4 (7.1)
Spain	17 (30.4)
France	11 (19.6)
United Kingdom	8 (14.3)
Netherlands	7 (12.5)
Czech Republic	3 (5.4)
Germany	2 (3.6)
Sweden	1 (1.8)
Israel	1 (1.8)
Portugal	1 (1.8)
Belgium	1 (1.8)
Prior HCT	
Auto HCT	19 (33.9)
Allo HCT	4 (7.1)
None	33 (58.9)
CAR T-cell product costimulatory domain	
CD28	22 (39.3)
41BB	27 (48.2)
CD28 and 41BB	3 (5.4)
Missing	4 (7.1)
Lymphodepleting chemotherapy	
Fludarabine/cyclophosphamide	53 (94.6)
Bendamustine	0 (0.0)
No LD chemo	0 (0.0)
Other	1 (1.8)
Unknown	2 (3.6)
Cytokine Release Syndrome (CRS) maximum grade	
No	15 (26.8)
1	22 (39.3)
2	15 (26.8)
3	3 (5.4)
4	0 (0.0)
Missing	1 (1.8)
Immune effector cell-associated neurotoxicity syndrome (ICANS) maximum grade	
No	45 (80.4)
1	4 (7.1)
2	4 (7.1)
3	2 (3.6)
4	0 (0.0)

Missing	1 (1.8)
Lung disease (BOS) before COVID-19	
Yes	1 (1.8)
No	45 (80.4)
Missing	10 (17.9)
Other lung pathology for example BOOP	
Yes	8 (14.3)
No	47 (83.9)
Missing	1 (1.8)
Symptoms during COVID-19	
Asymptomatic	7 (12.5)
Fever	36 (64.3)
Upper respiratory symptoms	29 (51.8)
Cough	32 (57.1)
Fatigue	22 (39.3)
Myalgia/Arthralgia	10 (17.9)
Vomiting	1 (1.8)
Diarrhea	8 (14.3)
Laboratory results at time of COVID-19	
Neutrophils (x10 9 /L)	2.2, 0.0 - 19.9 (48 pts)
Lymphocytes (x10 9 /L)	0.6, 0.0 - 2.4 (45 pts)
CD19 (cells x10^9)	0.0 (0.0 – 0.0) (4 pts)
CD3+/CD4+ (cells x10^9)	42.0, 25.0 - 192.0 (5 pts)
CD3+/CD8+ (cells x10^9)	56.0, 17.0 – 122.0 (5 pts)
IgG (g/l)	3.3, 0.4 - 18.0 (30 pts)
Platelets (x10 9/L)	121, 8 – 481 (49 pts)
CRP (mg/L)	27.5, 0.2 – 264.0 (40 pts)
Creatinine (mg/dl)	0.8, 0.2 - 3.0 (47 pts)
LDH level	256.0, 2.6 - 637.0 (39 pts)
Radiology CT scan	
Abnormalities Yes	38 (67.9)
Abnormalities No	6 (10.7)
Not performed	12 (21.4)
COVID-19 vaccination before COVID-19 infection	
Yes	2 (3.6)
No	43 (76.8)
Missing or unknown	11 (19.6)
COVID-19 vaccination after COVID-19	
Yes	7 (12.5)
No	38 (67.9)
Missing or unknown	11 (19.6)

Supplementary Table 2. Univariate analysis for factors associated with mortality

**Univariate analysis**			
		**Mortality HR (95% C.I.)**	**P**
**Variable**			
Age at time of COVID-19			
	10-year effect	1.44 (1.07-1.94)	0.015
	<60 years	1.00	
	>= 60 years	1.47 (0.67-3.23)	0.3
Sex			
	Male	1.00	
	Female	0.88 (0.40-1.97)	0.8
Time from CAR T-cell infusion to COVID-19			
	Continuous	0.94 (0.88-1.01)	0.1
	<6 months	1.00	
	>= 6 months	0.68 (0.31-1.51)	0.34
Metabolic comorbidity			
	No		
	Yes	2.78 (1.26-6.12)	0.01
Pre-existing lung pathology			
	No	1.00	
	Yes	1.99 (0.74-5.34)	0.17
CRS grade			
0		1.00	
>=1		2.32 (0.79- 6.78)	0.13
ICANS grade			
0		1.00	
>=1		0.66 (0.20-2.21)	0.5
Tumor remission status at time of COVID-19			
	CR	1.00	
	Other	2.46 (1.12-5.43)	0.026
COVID-19 treatment with convalescent plasma			
	No	1.00	
	Yes	0.62 (0.25-1.54)	0.3

In patients admitted to hospital only			
COVID-19 treatment with convalescent plasma			
	No		
	Yes	0.37 (0.15-0.93)	0.03
Wave			
	1st - Up to End of August 2020	1.00	0.5
	2nd - September 2020 - End of January 2021	0.61 (0.26-1.43)	
	3rd - From February 2021	0.56 (0.15-2.05)	
Performance status			
	10-point effect	0.74 (0.60-0.91)	0.004
